# Antimicrobial resistance of *Pseudomonas aeruginosa* isolated from patients with pneumonia during the COVID-19 pandemic and pre-pandemic periods in Northeast Brazil

**DOI:** 10.1590/1414-431X2023e12726

**Published:** 2023-07-21

**Authors:** G.P. Mesquita, M.C.C. Costa, M.A. Silva, L.G. Araújo, B.G. Vila Nova, É.J.M. Castro, L.C.M. Castelo Branco, R.C.S. da Silva, S.G. Marques, A.G. Abreu

**Affiliations:** 1 Laboratório de Patogenicidade Microbiana, Universidade Ceuma, São Luís, MA, Brasil; 2 Programa de Pós-Graduação em Ciências da Saúde, Universidade Federal do Maranhão, São Luís, MA, Brasil; 3 Laboratório Cedro, São Luis, MA, Brasil

**Keywords:** Drug resistance, Pneumonia, Pseudomonas aeruginosa, COVID-19

## Abstract

Healthcare-related infections caused by resistant microorganisms are a severe public health problem and are becoming increasingly prevalent in the hospital environment, especially *Pseudomonas aeruginosa*. This work aimed to evaluate the resistance profile of *Pseudomonas aeruginosa* to antimicrobials before the COVID-19 pandemic and during the pandemic period. Bacteria strains were obtained from tracheal aspiration, sputum, and bronchoalveolar lavage for diagnosis and phenotypic characterization. Matrix assisted laser-desorption ionization-time of flight mass spectrometry (MALD-TOF MS) was used to identify strains. Automated Phoenix and VITEK^®^ 2 Compact system and the disc diffusion method were performed to determine the antimicrobial susceptibility profile. A total of 41,000 medical reports from adult patients with pneumonia were analyzed. Of these, 951 patients were positive for *P. aeruginosa*, of which 373 were related to the pre-pandemic period and 578 to the pandemic period. Older men (≥60 years) were more prevalent in both periods. *P. aeruginosa* strains were resistant to imipenem in both periods: 38.8 and 42.5%, respectively, followed by meropenem (34.2 and 39.2%), ciprofloxacin (33.6 and 36.7%), and levofloxacin (34.9 and 43.5%). Intensive care units had the highest percentage of affected patients (62 and 65%) compared with other sectors, with a prevalence of 71% in the public network before COVID-19 and 59% during the pandemic. Our data showed a prevalence of *P. aeruginosa* in elderly patients in both the pre-pandemic and pandemic periods. In addition, an increase in *P. aeruginosa* resistance to beta-lactams, quinolones, carbapenems, and cephalosporins was observed during the COVID-19 pandemic compared with the period before the pandemic, especially in ICUs.

## Introduction

Healthcare-associated infections (HAIs) caused by resistant microorganisms are becoming increasingly prevalent, especially in the hospital environment (1), considered a site for opportunistic and virulent pathogens. However, this resistance challenge has surpassed the limits of hospitals (2).

Some factors contribute to this reality, such as indiscriminate use of antimicrobials by the population, globalization, which facilitates the transmission of resistance mechanisms among bacteria in the world, inadequate access to medicines, preference for broad-spectrum antimicrobials, deficit in the training of professionals in the area, as well as the scarcity of epidemiological surveillance for the control of antimicrobial drugs (3).

Thus, HAIs are a severe public health problem since they have both social and financial implications, mainly due to longer hospitalization stay and higher morbidity/mortality associated with higher cost of assistance (4,5).

In the hospital environment, infections are acquired after admission and may manifest during a hospital stay or after discharge (2,6), directly affecting the hospitalization process, especially in intensive care units (ICU). This occurs because ICU patients are more susceptible to several infections due to extended hospital stays, more invasive procedures, and an immunocompromised system with multiple drug use and associated comorbidities (6).

According to the World Health Organization (WHO), about 700,000 deaths are caused by infections from multidrug-resistant bacteria, and it is estimated that by 2050 there will be about 10 million deaths per year related to multidrug resistance (7).

*Pseudomonas aeruginosa*, a Gram-negative, aerobic, and non-glucose-fermenting bacteria, stands out among the main multidrug-resistant bacteria. It appears in the form of isolated rods or pairs and has the peculiar characteristic of forming colonies with greenish pigmentation due to the production of pioacinin, a toxic substance produced by this pathogen that delays the growth of other bacteria (8).

*P. aeruginosa* is commonly associated with more severe disease because it is an opportunistic microorganism, especially for patients with compromised immune systems (8). It is among the bacteria most commonly associated with hospital-acquired infections and ventilator-associated pneumonia (VAP) (2,9), and according to the WHO, in 2017, *P. aeruginosa* was considered a critical priority pathogen resistant to carbapenems, due to its multidrug resistance (10).

Beta-lactam antimicrobials, aminoglycosides, fluoroquinolones, polymyxins, and colistin are used to treat infections caused by *P. aeruginosa* (11,12). However, the high incidence of infections associated with the indiscriminate use of antimicrobials has resulted in a growing resistance, becoming a serious public health problem worldwide and a challenge for professionals in the field of the management of clinical treatment.

Furthermore, pneumonia is the main health care-related infection and affects mainly patients with endotracheal intubation and mechanical ventilation (2,13). About 10 to 20% of these patients develop pneumonia with a high mortality rate (13). In this context, COVID-19 is an acute respiratory infection caused by SARS-CoV-2 (severe acute respiratory syndrome coronavirus 2) and directly associated with patients in critical condition (14). The COVID-19 pandemic led to massive use of antimicrobials, which may lead to increased antimicrobial resistance (15), although the process is not fully understood. In addition, ventilator use worsens the clinical condition of COVID-19 patients.

Thus, considering the increase in *P. aeruginosa* resistance to various antimicrobials and the high incidence of VAP, especially during the COVID-19 pandemic, this study aimed to evaluate the resistance profile of *P. aeruginosa* to antimicrobials in the pre-pandemic period and during the pandemic in public and private hospitals in São Luís, in order to contribute to the development of health strategies to control bacterial resistance.

## Material and Methods

The study was carried out in a clinical analysis laboratory in São Luís, Northeast Brazil, which provided its samples for convenience. Data from electronic medical reports of patients infected by *P. aeruginosa*, referring to the pre-pandemic period, from 2019 to March 2020, and the period during the pandemic, from April 2020 to December 2021, were analyzed.

Inclusion criteria were adult male and female patients (≥18 years old) diagnosed with pneumonia caused by *P. aeruginosa*, referred from emergency rooms, outpatient clinics, medical and surgical clinics, surgical centers, and ICUs of public and private hospitals. Tracheal aspirate, sputum, and bronchoalveolar lavage samples were evaluated. Patients from neonatal and pediatric ICUs were not included.

Clinical and laboratory data included were the date of biological sample collection, patient age, sex, type of biological sample, microorganism isolated, and the result of the antibiogram, following the inclusion criteria.

Positive culture isolation was performed using MacConkey chromogenic medium and blood agar plates (BioMérieux, France). Subsequently, the isolates were identified by matrix assisted laser-desorption ionization-time of flight mass spectrometry (MALDI-TOF MS). The mass spectra acquired for each bacterial strain were compared to those contained in the database using Biotyper 3.0 software (Bruker, USA). All strains were kept in Luria Bertani broth supplemented with 15% glycerol at -80°C.

The Kirby Bauer plate diffusion technique was used for antimicrobial susceptibility testing, in addition to the automated Phoenix and VITEK^®^ 2 Compact system (BioMérieux), according to the Brazilian Committee on Antimicrobial Susceptibility Testing (BrCAST).

All isolates were tested for their susceptibility to the following antibiotics: amikacin, aztreonam, cefepime, ceftazidime, ciprofloxacin, gentamicin, imipenem, levofloxacin, meropenem, piperacillin/tazobactam, and polymyxin B, among others.

Following Resolution No. 466/2012 of the National Health Council (CNS), this research was approved by the Research Ethics Committee of the CEUMA University (Process No. 2.221.431/2017).

The results are reported as mean and standard deviation and were submitted to analysis of variance (ANOVA) and chi-squared test using the GraphPad Prism software (USA), version 7. The 95% confidence interval (P<0.05) was used to compare the sensitivity profile of isolated samples, age, sex, sectors, and hospital networks.

## Results

This study analyzed 41,000 medical reports of adult patients (≥18 years) with pneumonia admitted to public and private hospitals. Of these, 951 patients were selected, 373 of which were from the pre-pandemic period and 578 from the pandemic period.

There was a significant difference for the sex variable, with the men predominating in both periods (60 and 63%, respectively) (P<0.001).

The most prevalent age group in the two periods was ≥60 years, corresponding to 64 and 53%. The frequency of elderly was significantly higher than that of the 18-40 age group in the pre-pandemic period (P<0.05) and significantly higher than that of the other age groups during the pandemic period (P<0.05) (Table 1).

**Table 1:** Origin of samples used for *P. aeruginosa* culture and sociodemographic profile of patients with pneumonia treated at public and private hospitals in São Luís, northeastern Brazil.

	Pre-pandemic period		COVID-19 pandemic period	
	n	%	n	%
Samples				
Tracheal aspirate	353^a^	95	554^b^	96
Bronchoalveolar lavage	12	3	12	2
Sputum	8	2	12	2
Total	373	100	578	100
Gender				
Male	225^c^	60	363^d^	63
Female	148	40	215	37
Total	373	100	578	100
Age (years)				
18-40	59	16	101	18
41-59	74	20	169	29
≥60	240^e^	64	308^f^	53
Total	373	100	578	100

Data were analyzed using ANOVA or chi-squared test. ^a^P<0.0001 compared with the other samples of the pre-pandemic period; ^b^P<0.0001 compared with the other samples of pandemic period; ^c^P<0.001 compared with females of the pre-pandemic period; ^d^P<0.001 compared with females of the pandemic period; ^e^P<0.05 compared with the 18-40 age group of the pre-pandemic period; ^f^P<0.05 compared with the other age groups of the pandemic period.

Among the patients with positive *P. aeruginosa*, significantly more samples (P<0.0001) were from tracheal aspirate, both in the pre-pandemic period (95%, n=353) and during the pandemic (96%, n=554) (Table 1).

The antimicrobial susceptibility profile of *P. aeruginosa* of the 951 strains was evaluated, and no significant difference was found for the antimicrobials evaluated. However, it was possible to observe that the strains of *P. aeruginosa* were resistant mainly to carbapenems (imipenem and meropenem) and quinolones (levofloxacin and ciprofloxacin), both in the pre-pandemic and pandemic periods. Resistance to imipenem was 38.9% before the pandemic and 42.5% in the pandemic period. The same occurred for meropenem, with an increase in resistance from 34 to 39%.

Resistance to ciprofloxacin increased from 33.6 to 36.7% in the same analyzed periods. *P. aeruginosa* also showed an increase in resistance to levofloxacin, representative of the fluoroquinolone class, from 34.9 to 43.5% during COVID-19 (Table 2).

**Table 2:** Susceptibility profile of *P. aeruginosa* to the main antimicrobials tested in the clinic.

Antimicrobial	Pre-pandemic period				COVID-19 pandemic period			
	n	S	I	R	n	S	I	R
Amikacin	370	333 (90.00%)	6 (1.62%)	31 (8.38%)	574	512 (89.20%)	11 (1.92%)	51 (8.89%)
Aztreonam	164	96 (58.54%)	12 (7.32%)	56 (34.15%)	185	101 (54.59%)	13 (7.03%)	71 (38.38%)
Cefepime	367	255 (69.48%)	27 (7.36%)	85 (23.16%)	573	256 (44.68%)	167 (29.14%)	150 (26.18%)
Ceftazidime	371	254 (68.46%)	11 (2.96%)	106 (28.57%)	574	271 (47.21%)	147 (25.61%)	156 (27.18%)
Ciprofloxacin	369	234 (63.41%)	11 (2.98%)	124 (33.60%)	564	251 (44.50%)	106 (18.79%)	207 (36.70%)
Gentamicin	371	296 (79.78%)	10 (2.70%)	65 (17.52%)	399	305 (76.44%)	16 (4.01%)	78 (19.55%)
Imipenem	368	47 (12.77%)	189 (51.36%)	132 (38.87%)	570	44 (7.72%)	284 (49.82%)	242 (42.46%)
Levofloxacin	284	174 (61.27%)	11 (3.87%)	99 (34.86%)	462	185 (40.04%)	76 (16.45%)	201 (43.51%)
Meropenem	369	227 (61.52%)	16 (4.34%)	126 (34.15%)	600	319 (53.17%)	46 (7.67%)	235 (39.17%)
Piperacillin/Tazobactam	359	266 (74.09%)	35 (9.75%)	58 (16.16%)	461	274 (59.44%)	50 (10.85%)	137 (29.72%)
Polymyxin B	20	17 (85.00%)	0 (0.00%)	3 (15.00%)	152	135 (88.82%)	0 (0.00%)	17 (11.18%)

S: susceptible; I: intermediate; R: resistant.

Regarding 3rd and 4th generation cephalosporins ceftazidime and cefepime, *P. aeruginosa* showed significant resistance rates in the pre-pandemic period: 28.6 and 23.2%, respectively. The highlight was polymyxin B, which increased from 0.8 to 2.9% during the pandemic. Therefore, of the 11 antimicrobials analyzed, 9 showed increased resistance during the pandemic. Levofloxacin and piperacillin/tazobactam showed the most significant variation between periods, with increases of 8.6 and 13.6%, respectively (Table 2).

However, it is still possible to have a safety margin with the use of amikacin, which bacteria showed sensitivity in both periods, before and during the pandemic, corresponding to 90 and 89.2%, gentamicin, equivalent to 79.8 and 76.4%, and polymyxin B, with 85 and 88.8% respectively.

**Figure 1:**
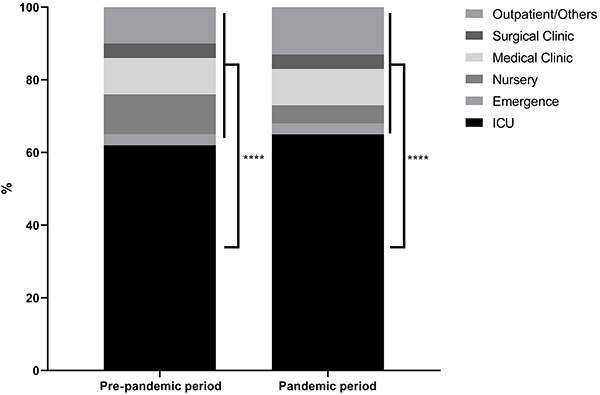
Distribution of *P. aeruginosa* in patients from different hospital sectors in the pre-pandemic and pandemic periods. Data are reported as mean. ****P<0.0001 (ANOVA).

The ICU was the sector with the highest number of affected patients, with a significant difference (P<0.0001) from the other sectors analyzed, reaching a percentage of 62% in the pre-pandemic period and 65% during the pandemic (Figure 1).

**Figure 2:**
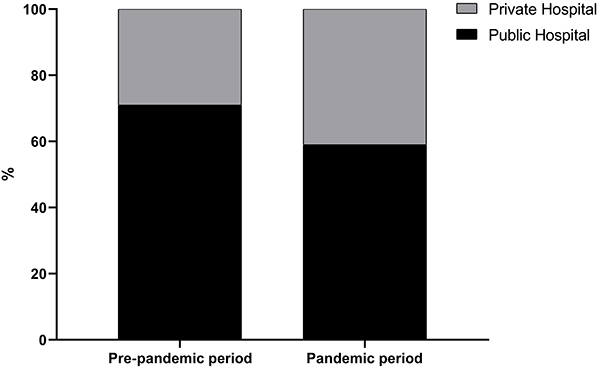
Type of hospital from which isolates were collected in the pre-pandemic period and during the pandemic in São Luis, Brazil. Data are reported as mean (ANOVA, P>0.05).

There was no statistical difference between the types of hospital networks, public and private, but most cases were from the public network both in the period before the pandemic (71%) and during the pandemic (59%). However, it is vital to note that during the pandemic, there was an increase in the number of hospitalizations in the private healthcare network from 29 to 41% among the evaluated hospitals (Figure 2).

**Figure 3:**
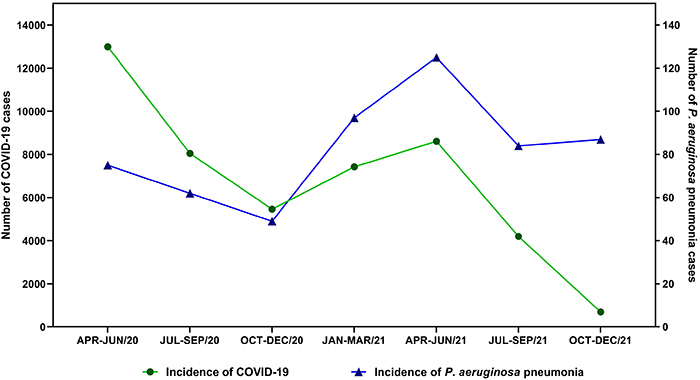
Relationship between the incidence of COVID-19 and *P. aeruginosa* pneumonia from April 2020 to December 2021.

Comparing COVID-19 infections with *P. aeruginosa* infections, it is possible to observe a descendant curve from April to December 2020 for both infections, with a progressive and continuous increase from January 2021, reaching the maximum point between April and June 2021. Thus, both infections had proportional increases in incidence, as there was an increase in COVID-19 cases in the population. Consequently, there were more infections and co-infections by *P. aeruginosa* (Figure 3).

**Figure 4:**
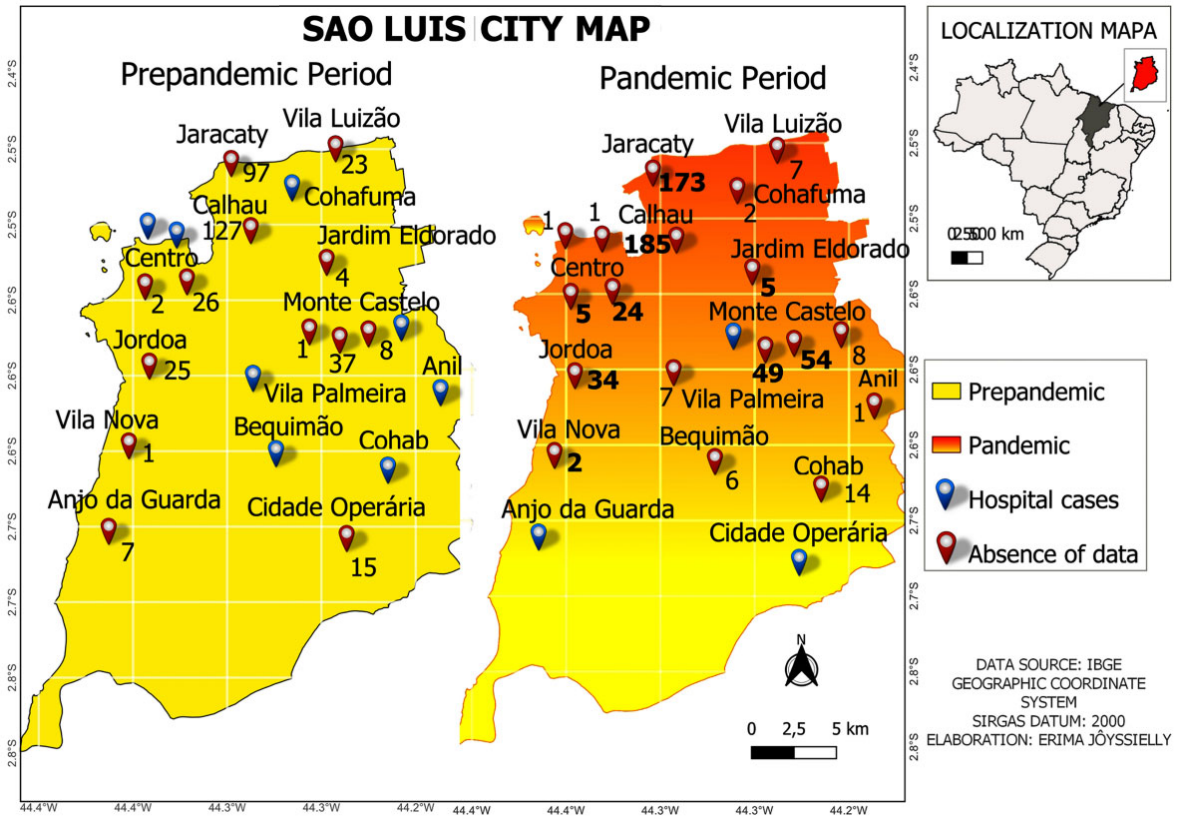
Heatmap of the incidence of pneumonia in hospitals in São Luis, Brazil, before the COVID-19 pandemic and during the pandemic.

There was an increase in pneumonia from *P. aeruginosa* from 97 to 173 cases in Jaracaty district and from 127 to 185 in Calhau before and during the pandemic (Figure 4). These numbers reflect the increase in the number of cases in hospitals during the pandemic.

## Discussion

In this study, we showed a significant increase in *P. aeruginosa* infections during the pandemic compared to before, especially in male patients aged ≥60 years compared to the other age groups. Some studies showed no difference between the sexes and had a higher prevalence of those over 60 years of age, as demonstrated in studies conducted in Goiás, Brazil (16) and Belém, North of Brazil, that showed a predominance of males (68%) compared to females (32%) in an oncology hospital (17).

On the other hand, a study that evaluated samples of ICU-related infections in the same city as our study showed that the women were more prevalent (50.4%) (18). This indicates that sex distribution can vary in a city in different periods. In addition, this study showed that the age group above 65 years (37.1%) was the most prevalent. Thus, age is a determining and facilitating factor for colonization and infection of this microorganism in pneumonia, as these patients have a more fragile physiological condition, which is susceptible to various infections (19).

Most samples in the study were from tracheal aspirates. According to a study in Paraná, southern Brazil, *P. aeruginosa* was most commonly found in tracheal aspirates, accounting for 36% (20). This is because the tracheal aspirate method is cheaper and therefore more accessible in places with few resources (21).

The use of mechanical ventilation through orotracheal intubation or tracheostomy in ICU patients favors susceptibility to major infections, given that these patients are immunocompromised (20,22).

VAP remains one of the most common infections in patients requiring invasive mechanical ventilation (22). According to a study in Wuhan, China, VAP occurred in 31% of patients with COVID, requiring invasive mechanical ventilation. Of these, 50% died from co-infections, which also led to an increase in nosocomial infections, generating a collapse in the healthcare system (23).

As in our study, an increase in microbial resistance of Gram-negative bacteria, such as *P. aeruginosa*, was observed in nosocomial infections in times of COVID-19, mainly for ceftazidime/avibactam, carbapenems, and polymyxin B, in another study in Fortaleza, northeastern Brazil (12).

Furthermore, a study that evaluated the sensitivity and resistance profile of bacteria isolated from tracheal aspirates from ICU patients between 2017 and 2018 showed an increase in resistance of *P. aeruginosa* to meropenem (from 20 to 46%), imipenem (from 40 to 45%) and piperacillin/tazobactam, which was not resistant in 2017, but reached 23% of resistant isolates in 2018, in addition to other antimicrobials such as ceftazidime, cefepime, and ciprofloxacin (24). These data are similar to our research, that showed an increase in resistance for the same antibiotics evaluated in bacteria isolated from patients with similar profiles.

A study conducted in Iran showed that 2.5% of *P. aeruginosa* isolates were resistant to colistin and polymyxin B, which has already been discussed and observed as a future problem since this is the last treatment option for critically ill patients (25). Importantly, in our study, there was an increase in the number of strains with resistance to polymyxin B, going from three strains in the pre-pandemic period to 17 in the COVID-19 pandemic period.

Cephalosporins are crucial in the treatment of patients with *P. aeruginosa* infection. However, many isolates of these bacteria are resistant to β-lactams, complicating the treatment of infections and leading to worse outcomes (26). Thus, carbapenems such as imipenem and meropenem are considered a therapeutic alternative to treat *P. aeruginosa* infections, although an alarming increase in resistance has been observed for such drugs, especially with their indiscriminate use in a hospital environment (27). In addition, an increase in microorganisms resistant to carbapenems during the pandemic was noted, confirming the increase in resistance in the last few years (28).

It is important to note that during the pandemic, resistance increased mainly because *P. aeruginosa* pneumonia often occurred as a co-infection in patients with COVID-19 (14), most of whom came from ICUs and were diagnosed as infected. It was not possible to perform a COVID-19 patient survival analysis since we did not have access to clinical data or patients' clinical outcomes, which was a limitation of this study.

Increasing resistance results from the widespread use of antimicrobials to control a disease with a previously unknown therapeutic agent, which may have influenced this increase in resistance to control a disease until then with an unknown therapeutic. Patients with a more extended hospital stay, especially in the ICU, tend to use more potent classes of antibiotics and in greater numbers due to the clinical situation. In our study, most isolates were from the ICU, both from private and public hospitals. As reported by another author, *P. aeruginosa* was more frequent in ICUs, accounting for 42.86% of cases (17). As in our study, a study in Pernambuco, northeast Brazil, demonstrated an increase in hospitalized patients with *P. aeruginosa* isolated from tracheal secretions in ICU in the periods evaluated (24).

Furthermore, SARS-CoV-2 has a tropism for upper and lower airways. Consequently, mechanical ventilation is frequently used in critically ill COVID-19 patients to promote respiratory muscle rest and allow adequate oxygen supply to tissues (29).

Most cases of nosocomial pneumonia are caused by bacteria that enter the pulmonary tree by aspiration from the oropharynx or gastric contents (30). Patients with severe SARS-CoV-2 infection who develop acute respiratory distress syndrome (ARDS) are thought to be at greater risk of acquiring these infections due to prolonged time on mechanical ventilation, confirming data showing a proportional relationship between the increase in incidence of COVID-19 and pneumonia caused by *P. aeruginosa*.

Understanding the bacterial resistance profile in our region is essential due to the lack of data and the constant increase of infections associated with antimicrobial resistance. Therefore, this study provides essential information on the impact of antibiotic resistance to improve surveillance of antibiotic-resistant infections, prevent and control the spread of bacterial resistance, strengthen policies and programs, and contribute to the implementation of prevention measures in the community and in hospitals.

Our data showed an increase in male patients with *P. aeruginosa* isolates isolated from the tracheal aspirate from the pre-pandemic to pandemic periods, with a predominance of older people (≥60 years). In addition, increased resistance of *P. aeruginosa* to carbapenems, cephalosporins, quinolones, and beta-lactams during the COVID-19 pandemic, especially in ICUs, was observed.
